# Thermodynamic stability and critical points in multicomponent mixtures with structured interactions

**DOI:** 10.1103/physrevresearch.4.033144

**Published:** 2022-08-22

**Authors:** Isabella R. Graf, Benjamin B. Machta

**Affiliations:** Department of Physics, Yale University, New Haven, Connecticut 06511, USA; Department of Physics and Quantitative Biology Institute, Yale University, New Haven, Connecticut 06511, USA

## Abstract

Theoretical work has shed light on the phase behavior of idealized mixtures of many components with random interactions. However, typical mixtures interact through particular physical features, leading to a structured, nonrandom interaction matrix of lower rank. Here, we develop a theoretical framework for such mixtures and derive mean-field conditions for thermodynamic stability and critical behavior. Irrespective of the number of components and features, this framework allows for a generally lower-dimensional representation in the space of features and proposes a principled way to coarse-grain multicomponent mixtures as binary mixtures. Moreover, it suggests a way to systematically characterize different series of critical points and their codimensions in mean-field. Since every pairwise interaction matrix can be expressed in terms of features, our work is applicable to a broad class of mean-field models.

Determining the phase behavior of mixtures is an important goal of statistical physics. However, while the thermodynamics of mixtures with few components is well understood theoretically [1], most functional mixtures are made up of a large number of distinct components, and the principles underlying the phase and critical behavior of such multicomponent mixtures are less clear. There have been substantial steps towards understanding these systems, but only in limiting cases. Sear and Cuesta [2] and subsequent follow-ups [3,4] determined conditions for phase separation in idealized mixtures with random, independent pairwise interactions. Taking a very different limit, Sollich and co-workers [5–7] have made progress for polydisperse mixtures interacting through a continuous distribution of attributes.

Different from these theoretical studies, many physical examples are made up of defined components whose interaction structure is governed by the physical details that underpin them. In phase-separation-prone lipid membranes, though there are thousands of chemical species, interactions are thought to be primarily driven by just a few features—interactions between headgroups, the degree of acyl-chain saturation, and the mismatch between hydrophobic heights [8–12]. In protein condensates, interactions are likely mediated by a combination of specific motifs, such as repetitive binding domains, and less specific electrostatic and hydrophobic interactions [13–15]. This observation suggests that in both cases the resulting effective pairwise interaction matrix is nonrandom in a particular way: Its rank, given by the number of independent features, can be considerably smaller than its dimension, given by the number of components. In other examples it might be less clear what features mediate interactions, but an effectively low-rank interaction matrix is likely common to most mixtures made up of many components. One class of examples are fluids such as petroleum for which an approximation in terms of lower-dimensional interaction parameters has been successfully applied [16].

To systematically investigate the role of such a low-dimensional interaction structure for phase behavior, in this paper we develop a theoretical framework to study the phase behavior of mixtures with many components but structured interactions. We show that the stability of phases and critical behavior can be understood in a “feature space,” which is typically much lower dimensional than the space of component densities.

## I. MEAN-FIELD MODEL

**FIG. 1.**
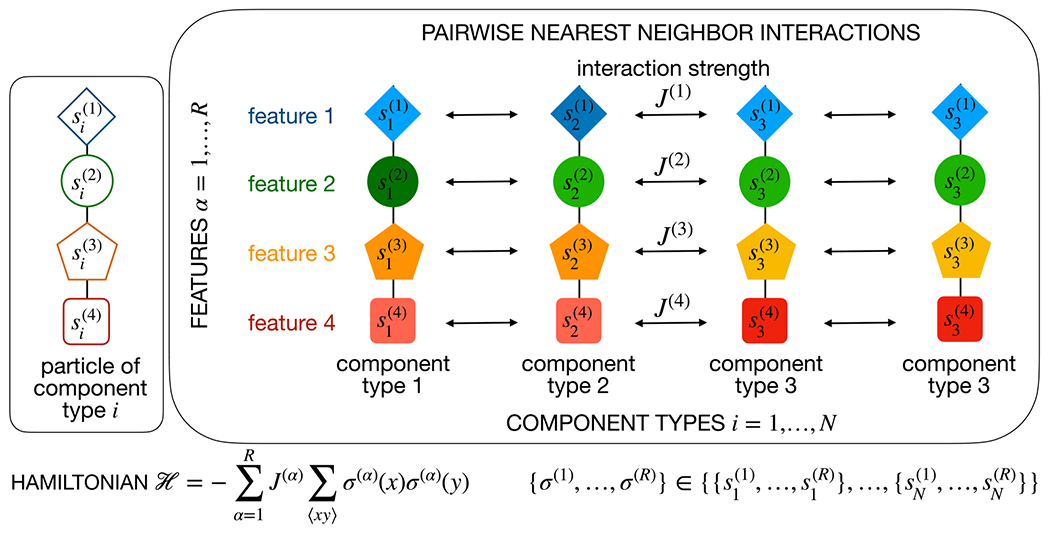
Model: The multicomponent mixture comprises N different component types (“components”). Component i=1,…,N is characterized by R features $s_{i}^{\left(\alpha \right)},\alpha =1,\ldots ,R$, each of which conveys an additive pairwise interaction with interaction strength $J^{\left(\alpha \right)}$ between neighboring components.

We specifically consider a family of multicomponent models with a pairwise interaction matrix of variable rank (see Fig. 1). The mixture is made up of N different component types. Component type i is characterized by a “feature vector” ${\vec{s}}_{i}$ composed of R real features $s_{i}^{\left(\alpha \right)},\;\alpha =1,\ldots ,R$. Each feature conveys an additive, Ising-like interaction with interaction strength $J^{\left(\alpha \right)}\neq 0$ [17]. The corresponding lattice Hamiltonian reads $𝒣=-\sum_{\alpha =1}^{R}\;J^{\left(\alpha \right)}\sum_{\left\langle xy\right\rangle }\;\sigma^{\left(\alpha \right)}(x)\sigma^{\left(\alpha \right)}(y)$, where $\sum_{\left\langle xy\right\rangle }$ is the sum over all neighboring sites x,y on the lattice and the spins take the values $\sigma^{\left(\alpha \right)}(x)=s_{i}^{\left(\alpha \right)}\in R$ if site x is occupied by component type i (see Ref. [18] for a related model with a single feature). In a mean-field approximation, our system is described by a Flory-Huggins-like free energy density per $k_{B}T$ [19,20] $f_{N}=\sum_{i=1}^{N}\;\rho_{i}\;\ln\;\rho_{i}-\sum_{i,j=1}^{N}\;\rho_{i}\chi_{ij}\rho_{j}$, where the densities $\rho_{i}$ are subject to the incompressibility constraint $\sum_{i}\;\rho_{i}=1$. The interaction matrix is given by

(1) $$\chi_{ij}:=\overset{R}{\underset{\alpha =1}{\sum }}\frac{zJ^{\left(\alpha \right)}}{2k_{B}T}S_{i}^{\left(\alpha \right)}S_{j}^{\left(\alpha \right)}=:\overset{R}{\underset{\alpha =1}{\sum }}C^{\left(\alpha \right)}S_{i}^{\left(\alpha \right)}S_{j}^{\left(\alpha \right)},$$

for a lattice coordination number z. For R features, the interaction matrix is of rank r⩽R. While this decomposition into features may be motivated by the physics of interactions, any real and symmetric interaction matrix $\chi_{ij}$ can be decomposed in this way, with R⩽N, eigenvectors $s_{i}^{\left(\alpha \right)}$, and eigenvalues $C^{\left(\alpha \right)}{\left|s^{\left(\alpha \right)}\right|}^{2}$; see Ref. [16] for a related (eigen)decomposition in the context of petroleum. A (precise) way to think about the features is thus as eigenvectors of the interaction matrix. Furthermore, as long as interactions are pairwise and meaningfully described by mean-field theory, our choice of representing components in terms of additive Ising-like features is entirely general. For now, we assume all eigenvalues to be positive but discuss the general case in the SM [21] and briefly below.

## II. THERMODYNAMIC STABILITY AND CRITICAL POINTS

The main challenge in working with mixtures with N≫1 components is that they are embedded in a very high dimensional space of densities. We now develop an analytic framework, wherein the mixtures are instead represented in the corresponding, potentially much lower dimensional feature space. To this end, we use matrix inversion techniques and will then successively derive conditions for local thermodynamic stability and the occurrence of different series of critical points.

In general, the thermodynamic behavior of the multicomponent mixture is determined by the free energy landscape in the N-dimensional space of densities ${\vec{\rho }}^{\left(N\right)}$. Due to the incompressibility constraint, the densities are not independent, $\rho_{N}=1-\sum_{i=1}^{N-1}\;\rho_{i}$, leaving the free energy density f a function of N−1 densities and temperature. The mixture is (locally) thermodynamically stable if the Hessian matrix (i,j=1,…,N−1)

(2) $$H_{ij}:=\frac{\partial^{2}f}{\partial \rho_{i}\partial \rho_{j}}=\underset{=:K_{ij}}{\underset{︸}{\delta_{ij}\frac{1}{\rho_{i}}+\frac{1}{\rho_{N}}}}-\underset{:={\left(UU^{T}\right)}_{ij}}{\underset{︸}{\overset{R}{\underset{\alpha =1}{\sum }}r_{i}^{\left(\alpha \right)}r_{j}^{\left(\alpha \right)}}}$$

is positive definite. Here, $r_{i}^{\left(\alpha \right)}:=\sqrt{2C^{\left(\alpha \right)}}\left(s_{i}^{\left(\alpha \right)}-s_{N}^{\left(\alpha \right)}\right)$ is the rescaled and shifted feature vector. While in the presence of a solvent, component N is most straightforwardly associated with this solvent, all physical results are ultimately independent of the choice of reference point. At high temperatures (large T→ small $C^{\left(\alpha \right)}\to$ small $r^{\left(\alpha \right)}$), the system is dominated by entropy (H≈K), and all eigenvalues of the Hessian matrix are positive; the system is thermodynamically stable to perturbations which are local in composition. At lower temperatures, one or more of the eigenvalues can become negative, implying that the system can spontaneously lower its free energy by phase separating along the corresponding eigenvector. The boundary of local thermodynamic stability is called the spinodal. It corresponds to the submanifold of compositions and temperature where the smallest eigenvalue of the Hessian matrix is zero. Since a matrix is invertible if and only if all of its eigenvalues are nonzero, the spinodal is also the submanifold where the matrix becomes singular for the first time, starting from all positive eigenvalues. Here, we take advantage of the fact that H is the sum of a positive definite matrix K with inverse $K_{ij}^{-1}=\delta_{ij}\rho_{i}-\rho_{i}\rho_{j}$ and a lower-rank contribution $UU^{T}$ arising from interactions [Eq. (2)]. Analogously to Ref. [22], we use the Woodbury matrix identity [23] on K and $UU^{T}$ to invert H and find that the Hessian matrix is invertible if and only if $1-U^{T}K^{-1}U=:1-Cov$ is invertible; see SM [21]. Here, Cov is the covariance matrix between the (rescaled) features:

(3) $${\text{Cov}}_{\alpha \beta }={\langle r^{\left(\alpha \right)}r^{\left(\beta \right)}\rangle }_{\vec{\rho }}^{\left(N\right)}-{\langle r^{\left(\alpha \right)}\rangle }_{\vec{\rho }}^{\left(N\right)}{\langle r^{\left(\beta \right)}\rangle }_{\vec{\rho }}^{\left(N\right)},$$

where the averages are taken with respect to the probability measure given by the mixture composition ${\vec{\rho }}^{\left(N\right)}:\langle X\rangle_{\vec{\rho }}^{\left(N\right)}=$ $\sum_{i=1}^{N}\;\rho_{i}X_{i}$. The rank of the covariance matrix $R_{Cov}$ corresponds to the maximal number of linearly independent feature vectors, $R_{\text{Cov}}⩽R$ [21].

### Thermodynamic stability.

These results imply that the mixture becomes unstable when the largest eigenvalue of the covariance matrix $\lambda^{\left(1\right)}=1$. Importantly, this condition is independent of the number of component types, including as limits the two-component mean-field Ising model and the infinite-component limit as discussed in the context of polydisperse systems [5,24].

### Direction of instability.

In order to find the initial direction of phase separation at the spinodal, we next determine the eigenvector corresponding to eigenvalue 0 (1) of the Hessian (covariance) matrix: If 1−Cov is invertible, the inverse of the Hessian matrix is given by $H^{-1}=K^{-1}+K^{-1}U(1-$ Cov${)}^{-1}U^{T}K^{-1}$. Using the eigendecomposition of the covariance matrix in terms of its (descending) eigenvalues $\lambda^{\left(\gamma \right)},\;\gamma =1,\ldots ,R$, and corresponding orthonormal eigenvectors $V^{\left(\gamma \right)}$ (whose dependency on ${\vec{\rho }}^{\left(N\right)}$ we drop for conciseness), ${Cov}_{\alpha \beta }=\sum_{\gamma =1}^{R}\;\lambda^{\left(\gamma \right)}V_{\alpha }^{\left(\gamma \right)}V_{\beta }^{\left(\gamma \right)}$, the inverse of the Hessian is $H_{ij}^{-1}=\delta_{ij}\rho_{i}-\rho_{i}\rho_{j}+\sum_{\gamma =1}^{R}\;\frac{1}{1-\lambda^{\left(\gamma \right)}}e_{i}^{\left(\gamma \right)}e_{j}^{\left(\gamma \right)}$, with

(4) $$e_{i}^{\left(\gamma \right)}:=\rho_{i}E_{i}^{\left(\gamma \right)}:=\rho_{i}\overset{R}{\underset{\alpha =1}{\sum }}V_{\alpha }^{\left(\gamma \right)}\left(r_{i}^{\left(\alpha \right)}-{\langle r^{\left(\alpha \right)}\rangle }_{\vec{\rho }}^{\left(N\right)}\right).$$

**FIG. 2.**
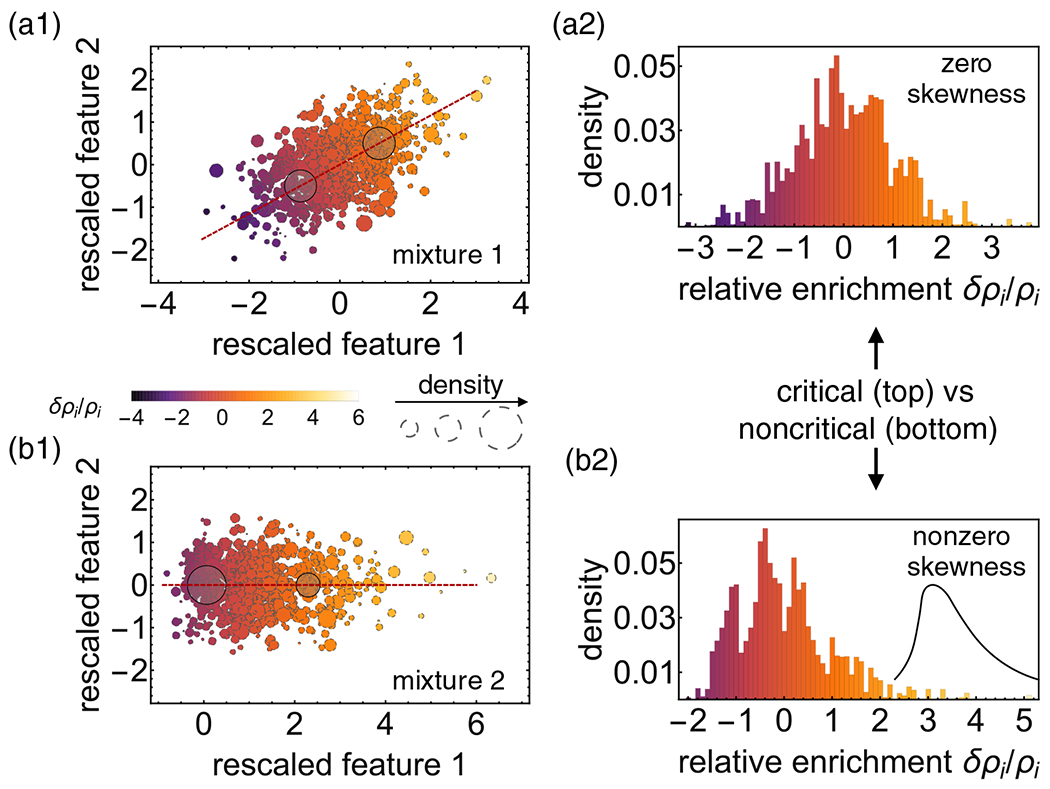
(a1) and (b1) Illustration of two multicomponent mixtures with N=1000 different component types (small, colorful disks; area of disk_*i*_ ∼ density $\left.\rho_{i}\right)$ in (R=2)-dimensional feature space, together with the first principal component (first PC; red dashed line) of their respective covariance matrix Cov. Both mixtures exhibit a variance of 1 along the first PC and are thus located on the spinodal. The relative enrichment $\delta \rho_{i}/\rho_{i}$ of component i=1,…,1000 along the initial direction of phase separation right at the spinodal (color code; arbitrary units) is determined by the projection $E_{i}^{\left(1\right)}$ of the feature vector (relative to the mean) onto the first PC of Cov; see Eq. (4). Coarse-graining the multicomponent mixture as an effective binary mixture that preserves the location of the system with respect to the spinodal and critical manifold (by conserving the second and third cumulant along the first PC of Cov) leads to a composition as shown by the large, translucent disks; see SM [21] for details. For a critical mixture (a), the composition of the binary mixture is symmetric, for a noncritical one (b), the densities of the two components are different. Note that all results are independent of global rotations or translations of the feature vectors or reflections $s_{i}^{\left(\alpha \right)}\to -s_{i}^{\left(\alpha \right)}\forall i$ [21]. (a2) and (b2) For the multicomponent mixture to be critical, the skewness of the distribution of relative enrichments has to be zero [mixture 1 in (a1) vs mixture 2 in (b1)]; see Eq. (5).

Close to the spinodal $\left(\lambda^{\left(1\right)}\approx 1\right)$, the dominant term is $\frac{1}{1-\lambda^{\left(1\right)}}e^{\left(1\right)}{\left(e^{\left(1\right)}\right)}^{T}$. Correspondingly, on the spinodal, $e^{\left(1\right)}$ is the eigenvector of the Hessian with eigenvalue 0 [25] and coincides with the direction of instability $He^{\left(1\right)}=0$ (see also Ref. [5] for polydisperse systems). Equation (4) implies that the relative enrichment $\delta \rho_{i}/\rho_{i}∼e_{i}^{\left(1\right)}/\rho_{i}=E_{i}^{\left(1\right)}$ of component i along the initial direction of phase separation at the spinodal (“partition coefficient”) is given by the deviations of the features from their mean, projected onto the first principal component (PC) of the feature distribution; see Fig. 2.

### Ordinary critical points.

The spinodal marks the edge of local thermodynamic stability. Except at special points, the spinodal lies within the binodal, the region of global thermodynamic stability. Points where the spinodal and binodal make contact are critical points (cp’s). At a usual critical point ${\vec{\rho }}^{\left(cp\right)}$, two phases become indistinguishable, corresponding to two minima and one maximum of the tilted Landau free energy $f\to f-{\left.\sum_{i}\;\rho_{i}\partial_{i}f\right|}_{cp}$ merging into one minimum. This merging occurs when the change in free energy along the direction of instability $\delta f=f\left(\rho^{\left(cp\right)}+\epsilon e^{\left(1\right)}\right)-f\left(\rho^{\left(cp\right)}\right)$ is zero up to order ⩽3 in ϵ. The first-order term of the tilted free energy is zero by definition, and ${\left.\left(\partial_{i}\partial_{j}f\right)e_{i}^{\left(1\right)}\right|}_{cp}=0$ as ${\vec{\rho }}^{\left(cp\right)}$ lies on the spinodal, yielding the following additional condition for the critical point:

(5) $${\left(\partial_{i}\partial_{j}\partial_{k}f\right)e_{i}^{\left(1\right)}e_{j}^{\left(1\right)}e_{k}^{\left(1\right)}|}_{cp}=0\to {\langle (E^{\left(1\right)}{)}^{3}\rangle }_{cp}=0,$$

where the average is with respect to the density at the critical point ${\vec{\rho }}^{\left(cp\right)}$; see SM [21] (compare also Ref. [5]). Thus the third cumulant (skewness) of the partition coefficient needs to be zero at a critical point; see Fig. 2. This condition on the third cumulant extends and substantiates the notion that binary systems and systems composed of ideal random copolymers are critical if their mixture composition is symmetric [26,27].

Notably, the conditions themselves $\left(\lambda^{\left(1\right)}=1\right.$ on spinodal, skewness =0 at a critical point) are valid irrespective of the mixture composition or feature distribution. To illustrate this generality, for Fig. 2 we randomly generated the components’ features (R=2) either via a multivariate Gaussian with zero mean [Figs. 2(a1) and 2(a2)] or as two independent features following a Poisson distribution (plus Gaussian noise) with nonzero mean and a Gaussian, respectively [Figs. 2(b1) and 2(b2)]. In both cases, the mixture composition is drawn from a uniform distribution over the N−1 simplex [21]. This procedure results in interaction matrices of rank 2, while “usual” random matrices have full rank [28].

### Higher-order critical points.

At an nth-order critical point ${\vec{\rho }}^{\left(cp\right)},n$ phases become indistinguishable. For a single order parameter (density) ρ, this condition corresponds to the merging of n minima and n−1 maxima into a single minimum of the tilted Landau free energy. The free energy expansion around the critical point is then of the order $2n:\delta f∼O\left(\delta \rho^{2n}\right)$. In a high-dimensional density space, the phases that become indistinguishable when crossing the nth-order critical point ${\vec{\rho }}^{\left(cp\right)}$ do not necessarily lie on a straight line. Instead, the phases merge along a more general smooth curve $\rho_{i}(\epsilon )=\rho_{i}^{\left(cp\right)}+\delta \rho_{i}(\epsilon )$ in density space, parametrized by ϵ [25]; see also Ref. [5]: $\delta \rho_{i}(\epsilon )=\sum_{m=1}^{\infty }\;\frac{\epsilon^{m}}{m!}\Upsilon_{i}^{\left(m\right)}$, for some vectors ${\vec{\Upsilon }}^{\left(m\right)},m\in N$, with $\sum_{k}\;{\vec{\Upsilon }}_{k}^{\left(m\right)}=0$ to conserve the incompressibility constraint. The (tilted) free energy change $\delta f(\epsilon )=f\left({\vec{\rho }}_{c}+\delta \vec{\rho }(\epsilon )\right)-$ $f\left({\vec{\rho }}_{c}\right)$, whose first-order term vanishes, should be of order 2n in $\epsilon :\delta f(\epsilon )={\left.\sum_{k=2}^{\infty }\;\frac{1}{k!}\sum_{i_{1},\ldots ,i_{k}}\;\frac{\partial^{k}f}{\partial \rho_{i_{1}}\cdots \partial \rho_{i_{k}}}\right|}_{cp}\delta \rho_{i_{1}}\cdots \delta \rho_{i_{k}}=$ $O\left(\epsilon^{2n}\right)$. At the same time, the system has to be stable against fluctuations in orthogonal directions. Thus we determine the curve $\delta \vec{\rho }$ around ${\vec{\rho }}^{\left(cp\right)}$ in a way that it minimizes the free energy change δf up to the respective order [21]. Systematically minimizing and setting the coefficients in front of $\epsilon^{m}$ to zero, we find the following conditions for an nth-order critical point in terms of the partial exponential Bell polynomials $B_{m,l}$ [21]:

(6) $$\frac{1}{2}\overset{R}{\underset{\alpha =1}{\sum }}\overset{m-1}{\underset{k=1}{\sum }}\left(\begin{array}{c} m\\ k \end{array}\right){\langle r^{\left(\alpha \right)}{\text{Ω}}^{\left(k\right)}\rangle }_{cp}{\langle r^{\left(\alpha \right)}{\text{Ω}}^{\left(m-k\right)}\rangle }_{cp}=\overset{m}{\underset{l=2}{\sum }}{\left(-1\right)}^{l}\left(l-2\right)!{\langle B_{m,l}\left({\text{Ω}}^{\left(1\right)},{\text{Ω}}^{\left(2\right)},\ldots \right)\rangle }_{cp},2⩽m⩽2n-1,$$

which only depend on the vectors $\Upsilon^{\left(m\right)}=:\rho^{\left(cp\right)}\Omega^{\left(m\right)},m=$ 1,…,n−1, determined recursively, namely, $\Omega^{\left(1\right)}=E^{\left(1\right)}$ and

$$\Omega^{\left(m\right)}=\overset{m}{\underset{l=2}{\sum }}\left[{\tilde{B}}_{m,l}-{\langle {\tilde{B}}_{m,l}\rangle }_{cp}+\overset{R}{\underset{\alpha =2}{\sum }}\frac{E\left(\alpha \right)}{1-\lambda^{\left(\alpha \right)}}{\langle E^{\left(\alpha \right)}{\tilde{B}}_{m,l}\rangle }_{cp}\right],$$

where ${\tilde{B}}_{m,l}:=(-1{)}^{l}(l-1)!B_{m,l}\left(\Omega^{\left(1\right)},\ldots ,\Omega^{\left(m-l+1\right)}\right)$. In the case of a single feature, R=1, this recursion is solved by $\Omega^{\left(m\right)}={\left.\partial_{\epsilon }^{m}\left(e^{\epsilon \sqrt{2C}s}/{\left\langle e^{\epsilon \sqrt{2C}s}\right\rangle }_{cp}\right)\right|}_{\epsilon =0}$, and the conditions for an nth-order critical point reduce to $\kappa_{2}^{\left(s\right)}=k_{B}T/(zJ)$ (spinodal) together with $\kappa_{m}^{\left(s\right)}=0\;\forall m=3,\ldots ,2n-1$. Here, $\kappa_{m}^{\left(s\right)}$ is the mth cumulant of the spin s with respect to ${\vec{\rho }}^{\left(cp\right)}$. Thus the more cumulants (order m⩾3) of the spin distribution are zero, the more phases become indistinguishable and the higher the order of the critical point can be. Merging of phases necessarily happens along the single direction of instability, and there is only one series of higher-order critical points—the one just discussed. This is not true for R>1, which we discuss next.

### Multiple directions of instability.

A series of critical points distinct from the previously discussed higher-order critical points occurs when the largest eigenvalue 1 of Cov is D-fold degenerate [21]. To ensure stability along any direction in the corresponding D-dimensional subspace of eigenvectors, the third cumulant of all vectors in the subspace then needs to equal 0. For example, a system has a critical point with two unstable directions if it has a twofold degenerate maximal eigenvalue, $\lambda^{\left(1\right)}=\lambda^{\left(2\right)}=1$, and if the four distinct third cumulants $\kappa^{\left(\alpha \beta \gamma \right)}:={\left\langle E^{\left(\alpha \right)}E^{\left(\beta \right)}E^{\left(\gamma \right)}\right\rangle }_{cp}$ with αβγ=111,112,122,222 are zero. In general, a (D+1)th-order critical point with D degenerate unstable directions has codimension $\left(\begin{array}{c} D+1\\ 2 \end{array}\right)+\left(\begin{array}{c} D+2\\ 3 \end{array}\right)$: It requires tuning $\left(\begin{array}{c} D+1\\ 2 \end{array}\right)$ parameters for Cov to have its D largest eigenvalues equal to 1 [29] and $\left(\begin{array}{c} D+2\\ 3 \end{array}\right)$ for the third cumulants [21].

The emergent symmetry of these critical points is reminiscent of the order-parameter symmetry in q-state Potts models, with q=D+1. In two dimensions there are known to be critical transitions in the q-state Potts model for q⩽4 [30], but in mean-field these transitions are first order [31,32] except for the case of the Ising model, q=2 [33,34]. Our results show that it may be possible to have critical transitions in mean-field models that have the symmetry of q-state Potts models, but only in models with sufficient flexibility. For q=3, an example of an N=6 component model with R=2 features is given explicitly in the SM [21].

## III. MODEL EXTENSIONS

So far we have focused on a mean-field free energy whose interaction matrix has positive eigenvalues and whose components are of the same size. We now briefly discuss how the previously discussed conditions generalize to the case of negative interaction strengths and comment on components with different sizes as in the original Flory-Huggins theory [19,20] in the SM [21]. If the feature interaction strengths $J^{\left(\gamma \right)}$ are all positive, the pairwise interactions satisfy $2\chi_{ij}-\left(\chi_{ii}+\chi_{jj}\right)<0\forall i,j$, and interactions between alike components are always energetically preferred compared with dislike components. To resolve this limitation, we consider the general case with $R^{+}$”positive” attractive features and $R-R^{+}$”negative” repulsive ones: $J^{\left(\alpha \right)}>0\forall \alpha =$ $1,\ldots ,R^{+}$ and $J^{\left(\alpha \right)}<0\forall \alpha =R^{+}+1,\ldots ,R$. Performing an analysis that is conceptually similar to but more intricate than that performed before, the spinodal criterion is $\lambda_{\overset{‾}{C}}^{\left(1\right)}=$ 1. Here, $\lambda_{\overset{‾}{C}}^{\left(1\right)}$ is the largest eigenvalue of the real, symmetric matrix $\overset{‾}{C}=C^{\left(++\right)}-C^{\left(+-\right)}{\left(1+C^{\left(--\right)}\right)}^{-1}C^{\left(-+\right)}$, which is determined by the covariances among the subsets of positive (+) and negative (−) features; see SM [21]. $\overset{‾}{C}$ has dimensions $R^{+}\times R^{+}$ and can be interpreted as representing a multicomponent system with $R^{+}$ positive features and effective, reduced interactions. The extent to which the negative features influence the phase behavior depends on the relative correlations between all features. If for each dominant positive feature, there is a highly correlated negative feature of similar strength, their effects will roughly cancel, and the mixture will not phase separate. Conversely, if the dominant positive features driving phase separation correlate weakly with the negative features, thermodynamic stability is barely modified by the presence of the latter. At the spinodal, the direction of instability is ${\overset{‾}{e}}_{i}^{\left(1\right)}=\rho_{i}\sum_{\alpha =1}^{R^{+}}\;\phi_{\alpha }^{\left(1\right)}\left[\pi_{i}^{\left(\alpha \right)}-\right.\;\left.\sum_{\beta ,\gamma =1}^{R-R^{+}}\;C_{\alpha \beta }^{\left(+-\right)}{\left(1+C^{\left(--\right)}\right)}_{\beta \gamma }^{-1}\nu_{i}^{\left(\gamma \right)}\right]$ in terms of the first eigenvector $\phi^{\left(1\right)}$ of $\overset{‾}{C}$ and the deviations of positive (π) and negative ((ν) features from the mean; see SM [21]. We observe that this direction of instability again corresponds to (a combination of) feature deviations from the mean, projected onto the first principal component $\phi^{\left(1\right)}$ (now of the “effective covariance matrix” $\overset{‾}{C}$). Roughly speaking, the relative sign of the contributions of the negative and positive features depends on whether they are correlated or anticorrelated (negative or positive sign). Finally, performing the same analysis as for the original model, we find an analogous condition for the ordinary critical point: ${\left\langle {\left({\overset{‾}{E}}^{\left(1\right)}\right)}^{3}\right\rangle }_{cp}=0$, where ${\overset{‾}{e}}_{i}^{\left(1\right)}=:\rho_{i}{\overset{‾}{E}}_{i}^{\left(1\right)}$.

## IV. DISCUSSION

In this paper, we consider a general mean-field model for multicomponent mixtures with an arbitrary pairwise interaction matrix $\chi_{ij}$ of variable rank which we decompose in terms of different “features” mediating additive interactions between the components. The analytic conditions we derive for the spinodal and (higher-order) critical points only depend on the distribution of components in feature space. Specifically, the spinodal and submanifold of ordinary critical points are determined exclusively by the variance and third cumulant of the component distribution projected along the first principal component of the feature covariance matrix (Fig. 2).

This representation in feature space is reminiscent of the dimensional reduction obtained for polydisperse systems whose excess free energy only depends on a few generalized moments of the attributes [5–7]. While the derivation of the “moment free energies” relies on either a division of density space into a subspace of moments and its “transverse” space or on combinatorial arguments [5,6], here we instead exploit the fact that the condition for the Hessian matrix to become singular only depends on an R-dimensional matrix originating from the interaction structure. A related simplification of the spinodal condition in terms of a lower-dimensional matrix has been achieved for Flory-Huggins models with an excess free energy depending only on a finite number of moments of the molecular weight distribution [35–37].

The representation in feature space also suggests a principled method for finding coarse-grained binary mixtures with similar properties. By choosing the composition and interaction strength of the binary mixture so as to preserve the second and third cumulant along the first principal component, the coarse-grained binary mixture maintains the location of the multicomponent system with respect to the spinodal and critical manifold; see Fig. 2 and SM [21].

In addition, our analysis allows for a systematic identification of the codimension of different series of critical points in multicomponent systems; see also Refs. [38,39]. For instance, we find that, in the absence of symmetries, a tricritical point has codimension four in mean-field. Furthermore, higher-order critical points with symmetry reminiscent of the q-state Potts model require tuning of $\left(\begin{array}{c} q\\ 2 \end{array}\right)+\left(\begin{array}{c} q+1\\ 3 \end{array}\right)$ parameters. For the (q=3)-states Potts model, this counting suggests a codimension of 7 for the critical point, which is larger than the one accessible with just N=3 components but feasible for a mean-field model with N=6 components and R=2; we explicitly construct such a (q=3)-states-Potts-like model containing a critical point [21].

Our results offer an appealing avenue towards understanding intracellular liquid-liquid phase separation [15] and the critical phase behavior observed in cell-derived plasma membranes [40]. These mixtures are composed of thousands of proteins (and lipids), and depending on the conditions, small domains form spontaneously. The number of coexisting domains appears to be orders of magnitude smaller than the number of components and is thus well below the limit set by Gibbs’s phase rule [41]. In cell-derived plasma membranes, while true phase separation occurs when cooling them below the critical temperature [42], nanoscopic domains observed at physiological temperatures [43] have been suggested to be critical fluctuations close to a thermodynamic critical point in the two-dimensional (2D) Ising universality class [40]. Strikingly, specific lipids and proteins robustly partition into specific phases—seemingly under fairly broad conditions [44]. Our work offers an interpretation of this experimental observation: Phase behavior is determined by just a few important features. Looking for such a low-dimensional feature space representation might help to make sense of the growing amount of experimental data generated by proximity-labeling techniques [45] and should provide important insights into the physical characteristics underlying intracellular phase separation. In these biological systems, effects of finite dimension (two or three) and sequence-dependent interaction patterns [46,47] will likely quantitatively, but not qualitatively change the mean-field picture we present here. Finally, our analytic theory only makes predictions about local thermodynamic properties but cannot now make statements about the global phase behavior, which would require knowledge of the full free energy landscape [1,3,4,48,49]. Whether global phase behavior can be understood in feature space is an interesting question for future research.
